# Culturomics Profiling of Nasal Cavities of European Wild Rabbits on the Iberian Peninsula: Antimicrobial Resistance and Detection of Microorganisms of Public Health Interest

**DOI:** 10.3390/pathogens14040317

**Published:** 2025-03-26

**Authors:** Carmen González-Azcona, Saúl Jiménez-Ruiz, Nuno Santos, Inés Del Campo-Fernández, Katherine Rojas-Tigasi, Tamara Álvarez-Gómez, Irene Marañón-Clemente, Paula Eguizábal, Idris Nasir Abdullahi, Carla Andrea Alonso, Carmen Torres, Carmen Lozano

**Affiliations:** 1 Area of Biochemistry and Molecular Biology, OneHealth-UR Research Group, University of La Rioja, 26006 Logroño, Spain; carmen.gonzalezaz@unirioja.es (C.G.-A.); ines.del-campo@alum.unirioja.es (I.D.C.-F.); katherine-andrea.rojas@alum.unirioja.es (K.R.-T.); tamara.alvarez@unirioja.es (T.Á.-G.); irene-olga.maranon@unirioja.es (I.M.-C.); paula.eguizabalm@unirioja.es (P.E.); eedris888@yahoo.com (I.N.A.); 2 Departamento de Sanidad Animal, Grupo de Investigación en Sanidad Animal y Zoonosis (GISAZ), UIC Zoonosis y Enfermedades Emergentes ENZOEM, Universidad de Córdoba, 14014 Córdoba, Spain; saul.jimenez.ruiz@gmail.com; 3 Centro Investigação Biodiversidade e Recursos Genéticos (CIBIO), Laboratório Associado (InBIO), Universidade do Porto, 4485-661 Vairão, Portugal; pygargusv@sapo.pt; 4 Department of Medical Laboratory Science, College of Medical Sciences, Ahmadu Bello University, Zaria 810107, Nigeria; 5 Servicio de Análisis Clínicos, Laboratorio de Microbiología, Hospital San Pedro, 26006 Logroño, Spain; caalonso@riojasalud.es

**Keywords:** wild rabbits, nasal colonization, *Staphylococcus*, *Enterococcus*, *Enterobacter*, Citrobacter, wildlife, resistance, surveillance

## Abstract

**Background:** European wild rabbits (*Oryctolagus cuniculus*) are closely connected to the natural environment and might be a potential source of pathogenic bacteria and/or antimicrobial-resistant bacteria. The objective was to identify the bacterial community (species and genera) that colonizes the nasal cavities of European wild rabbits as well as to study the antimicrobial resistance (AMR) phenotypes of bacteria of public health interest. **Methods:** A total of 147 nasal swabs individually collected from wild rabbits in Spain and Portugal (between 2022 and 2024) were studied. Samples were inoculated in different culture media, and isolates were identified by MALDI-TOF. The AMR phenotypes of staphylococci, mammaliicocci, enterococci and Enterobacterales were evaluated by the disk-diffusion method. **Results:** Overall, 557 non-repetitive isolates were obtained (1 isolate per species and AMR phenotype of each animal). A wide diversity of genera (n = 40) and species (n = 90) was found. *Staphylococcus* (21.2%), *Mammaliicoccus* (11.7%), *Enterococcus* (23.3%), *Enterobacter* (9.2%), *Citrobacter* (4.5%) and *Escherichia* (5.9%) were the most detected genera. Most animals presented more than one genera (78.9%), and in 15.7% of them, at least five genera were identified. Susceptibility to all antimicrobials tested was found in 37.2%, 38.5% and 51.6% of staphylococci/mammaliicocci, enterococci and *Escherichia coli* isolates; moreover, multidrug resistance was detected in 10.4%, 14.6% and 9.6% of these groups of bacteria. Moreover, important species of pathogenic bacteria were found, such as *Yersinia enterolocolitica* (0.5%) and *Bordetella bronchiseptica* (0.2%), among others. **Conclusions:** A high bacterial diversity was detected in the nasal cavities of European wild rabbits from the Iberian Peninsula, including pathogenic species and/or resistant strains of public health interest.

## 1. Introduction

Wildlife has been recognized to be an important link in One Health ecosystems, as it can be a source of microorganisms that could cause emerging and re-emerging infectious diseases that could spill over to urban settlements and infect humans and domestic animals [1]. In this regard, bacterial colonization of wild animals could be an indicator of environmental contamination or anthropogenic activities in the wild [2,3]. The management of wildlife health is closely linked to human and veterinary public health under a One Health context. In this sense, it is highly important to monitor and counteract the spread of antimicrobial resistance (AMR) in the natural environment [4,5].

Wildlife is not directly exposed to clinical antimicrobial agents but can acquire antimicrobial-resistant bacteria through contact with humans, animals and the contaminated environment [6]. Water polluted with feces appears to be the most significant source of contamination for wild animals [1]. This condition promotes the emergence and persistence of AMR, and wildlife commensal bacterial strains may become reservoirs of AMR genes [3,7]. Moreover, other factors could favor the presence of antibiotic-resistant bacteria in wildlife, such as the presence of resistance genes in mobile genetic elements or the emergence of spontaneous mutations [8]. AMR might be selected not only by the presence of antimicrobial agents but also by other chemical substances such as heavy metals and/or biocides [9]. To provide a broader picture of pathogenic bacteria in wildlife, prevalence studies across different habitats and animal species are necessary.

The European wild rabbit is an herbivore mammal of small size, native to the Iberian Peninsula, where two subspecies diverged around 1.8 million years ago [10]: *Oryctolagus cuniculus algirus* and *Oryctolagus cuniculus cuniculus*. *Oryctolagus c. algirus* is smaller and occurs in the southeastern Iberian Peninsula, North Africa, the Mediterranean and the Atlantic islands [11]. *Oryctolagus c. cuniculus* is larger and occurs in the northeastern Iberian Peninsula, France, and many other areas around the world where they were introduced by humans [11]. *Oryctolagus c. cuniculus* is also an ancestor of all domestic rabbits [12].

The European wild rabbit is an important species for hunting activities and can enter the food chain [13,14]. Moreover, it plays a fundamental role in the ecology of Iberian ecosystems and is also economically relevant, particularly in Europe [15]. The European wild rabbit might be a potential source of bacteria carrying relevant AMR genes for other domestic and wild animals, even for humans, because interactions may occur across different ecosystems [5,6,15]. The role of wild rabbits in infectious disease epidemiology is still uncertain. However, it is known that wild animals can act as reservoirs and could be sources of zoonotic diseases during anthropogenic activities in wildlife [16,17].

It has previously been observed that wild rabbits can host a wide variety of bacteria with AMR phenotypes of relevance in public health [18,19,20]. However, few studies have addressed the nasal microbiota of these animals [15,21]. In the present study, the main objective was to isolate and identify the bacterial community (species and genera) that colonizes the nasal cavities of European wild rabbits from Spain and Portugal as well as to study the AMR phenotypes of genera of public health interest.

## 2. Material and Methods

### 2.1. Sample Collection, Transport and Preservation

**Figure 1:**
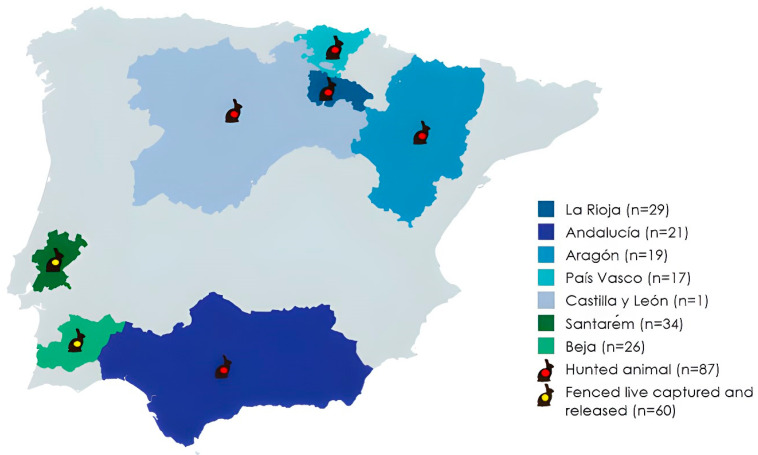
Geographical areas of the Iberian Peninsula in which the European wild rabbit samples were obtained and how animals were captured.

Nasal samples of 147 European wild rabbits were obtained in different areas of the Iberian Peninsula from November to December 2022, February 2023, and August 2024 (Figure 1). Sampling was as follows: a) 87 samples of wild rabbits from different regions of Spain (La Rioja, Aragón, País Vasco, Castilla y León and Andalucía) and b) 60 samples of wild rabbits from different regions of Portugal (Santarém and Beja). All samples from Spain were obtained during the game season from legally hunted animals. Portuguese rabbits were sampled both from hunting (n = 26; from the counties Ourique and Almeirim, in Satarém) and captures for a longitudinal capture–mark–recapture study (n = 8; at Parque de Natureza de Noudar, Beja) [22]. The study was approved by the ethical committee of the CIBIO’s Animal Welfare and Ethics Review Board (ORBEA/2023_01).

For nasal sampling, sterile cotton-tipped swabs were used and briefly inserted into the nostrils. They were immediately transferred to commercial tubes of Amies transport medium and stored at 4 °C until arrival at the laboratory for processing. In case they could not be processed in one or two days, they were frozen at −80 °C until laboratory analysis.

### 2.2. Bacterial Isolation and Identification

Nasal swab samples were placed in Brain Heart Infusion broth (BHI, Condalab, Madrid, Spain). Samples were inoculated in duplicate: one in BHI broth with 6.5% sodium chloride (NaCl) and the other one non-supplemented with NaCl. Both media were incubated for 24 h at 37 °C. After incubation, the broth samples were inoculated into five culture media. Broth samples supplemented with NaCl were inoculated on mannitol salt agar (MSA, Condalab, Madrid, Spain) and MRSA chromogenic media for the isolation of staphylococci (methicillin-susceptible and methicillin-resistant, respectively). The unsupplemented broth samples were inoculated on blood agar (BioMerieux, Madrid, Spain), MacConkey agar (OXOID, Madrid, Spain) and Slanetz Bartley agar (Scharlab, Sentmenat, Spain) for the isolation of Gram-positive and Gram-negative bacteria. Plates were incubated for 24 to 48 h at 37 °C for bacterial recovery. After overnight growth, up to 13 different colonies per sample (based on morphology, color and haemolysis) were randomly selected. Colonies were identified by matrix-assisted laser desorption/ionization time-of-flight mass spectrometry (MALDI-TOF; Bruker Daltonics, Bremen, Germany) using the standard extraction protocol recommended by the manufacturer. For calibration of the spectrometer, the protein profile of the DH5 peptide from the *Escherichia coli* strain was used [23].

### 2.3. Antimicrobial Susceptibility Testing

The antimicrobial susceptibility phenotypes were analyzed in all staphylococci, enterococci, *Escherichia coli*, *Klebsiella* spp. and *Yersinia enterocolitica* isolates obtained. For this purpose, the disk-diffusion method was used and the following antimicrobial agents were employed (charge of disk in mcg): (a) in staphylococci: penicillin (10), cefoxitin (30), erythromycin (15), clindamycin (2), gentamicin (10), tobramycin (10), tetracycline (30), ciprofloxacin (5), chloramphenicol (30), linezolid (30), trimethoprim–sulfamethoxazole (1.25 + 23.7) and mupirocin (200); (b) in enterococci: penicillin (10), vancomycin (30), teicoplanin (30), erythromycin (15), gentamicin (120), streptomycin (300), tetracycline (30), ciprofloxacin (5), chloramphenicol (30) and linezolid (30); and c) in Gram-negative bacteria: ampicillin (10), cefoxitin (30), amoxicillin/clavulanic acid (30), cefotaxime (30), ceftazidime (30), imipenem (10) gentamicin (10), tobramycin (10), tetracycline (30), ciprofloxacin (5), trimethoprim-sulfamethoxazole (25) and chloramphenicol (30). The staphylococcal disk diffusion results were interpreted according to the European Committee of Antimicrobial Susceptibility Testing and the enterococcal, *E. coli, Klebsiella* spp. and *Y. enterocolitica* susceptibility testing data according to the Clinical and Laboratory Standards Institute [24,25].

### 2.4. Statistical Analysis

A correlation matrix of bacteria species detected in European wild rabbits was formulated. To analyze the correlation between whole diversity, a graphic model was performed in R 4.4.1 (R Core Team, 2024) using the R packages ‘corrplot’ (0.9.5) and readxl (1.4.3).

## 3. Results

### 3.1. Isolates and Genera Detected in Nasal Samples

A total of 598 isolates were recovered. After AMR phenotyping, 557 non-repetitive isolates (1 isolate per species and antimicrobial resistance phenotype per animal) were selected for further analysis. The distribution of isolates from the 147 nasal samples from European wild rabbits was as follows: 291 isolates of 60 samples from Portugal and 266 isolates of 87 samples from Spain. A total of 40 genera and 90 species were identified. Overall, 344 isolates were Gram-positive, and there were 213 Gram-negative bacteria (Table 1 and Table 2). Among the Gram-positive bacteria, the most common genera were the following ones (number of isolates in brackets): *Enterococcus* (130), *Staphylococcus* (118) and *Mammaliicoccus* (65). For the Gram-negative bacteria, the most common genera were (number of isolates in brackets): *Enterobacter* (48), *Escherichia* (33) and *Citrobacter* (25).

### 3.2. Diversity of Genera and Species Detected

**Figure 2:**
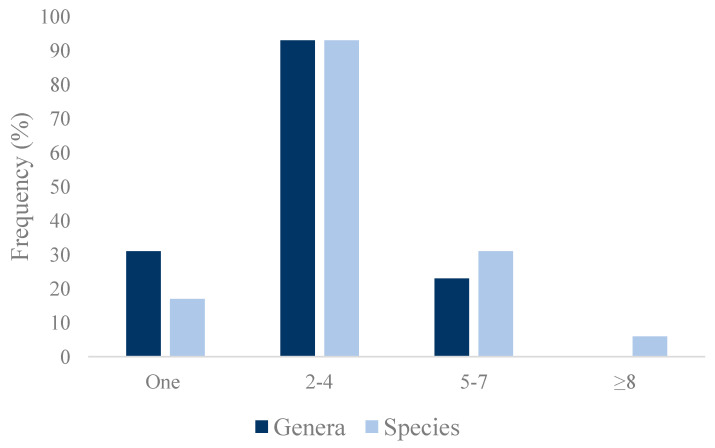
Frequency of bacterial genera and species identified in the 147 nasal samples from European wild rabbits.

In all 147 European wild rabbits, at least one bacterial isolate was obtained and identified. In general, a high diversity of genera and species was found. Most of the animals tested presented more than 1 genera (78.9%), and in 15.7% of them, ≥5 genera were identified. Seven different genera were obtained in four of the animals tested, and they were the following ones: *Achromobacter*, *Butiauxella*, *Citrobacter*, *Enterobacter*, *Enterococcus*, *Escherichia*, *Hafnia*, *Klebsiella*, *Kocuria*, *Lelliottia*, *Mammaliicoccus*, *Micrococcus*, *Raoultella*, *Stenotrophomonas*, *Staphylococcus* and/or *Streptococcus*. A single genus was recovered in only 31 of the rabbits (21.1%) (Figure 2).

Overall, 90 bacterial species (44 Gram-positive and 46 Gram-negative) were identified. Only 17 (11.6%) animals presented a single bacterial species. The remaining 130 European wild rabbits showed more than one, most of them containing from two to four species (63.3%). From 8 to 12 bacterial species were identified in six individuals (4.1%) (Figure 2). In the sample in which the highest diversity of species was detected (seven genera and 12 species), the following species were found: *Citrobacter freundii*, *Citrobacter gillenii*, *Enterococcus casseliflavus*, *Enterococcus faecium*, *Enterococcus hermanniensis*, *Enterococcus hirae*, *Enterococcus mundtii*, *Escherichia coli*, *Hafnia alvei*, *Klebsiella oxytoca*, *Mammaliicoccus lentus* and *Streptococcus parauberis*.

### 3.3. Diversity of Gram-Positive Bacteria

**Figure 3:**
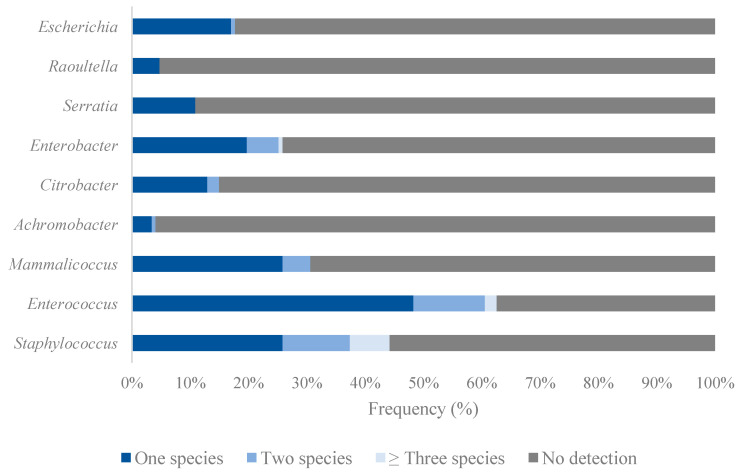
Frequency of bacterial species detected among the 147 European wild rabbits. Genera in which ≥3 different species were identified are represented.

Most of the animals were colonized by enterococcal (62.6% of the animals), staphylococcal (44.2%) and mammaliicoccal (30.6%) isolates, and a wide diversity of bacterial species (9 *Enterococcus* spp., 16 *Staphylococcus* spp. and 3 *Mammaliicoccus* spp.) was identified among these genera (Figure 3, Table 1). Two coagulase-positive staphylococci (CoPS) species were detected: *S. aureus* in 20 animals (13.6%) and *S. pseudintermedius* in 10 animals (6.8%) (Table 1). In addition, no staphylococci were recovered in 82 animals (55.8%) (Figure 3). In most of the nasal samples, only one staphylococcal species was identified (25.9%), and eight animals carried three staphylococcal species: *S. aureus* + *S. pseudintermedius* + *S. hyicus* or *S. xylosus*; *S. aureus* + *S. xylosus* + *S. saprophyticus* or *S. simulans*; *S. epidermidis* + *S. saprophyticus* + *S. gallinarum* or *S. xylosus*; *S. epidermidis* + *S. borealis* + *S. haemolyticus*; and *S. capitis* + *S. equorum* + *S. saprophyticus*. Two animals carried four staphylococcal species: *S. aureus + S. pseudintermedius* + *S. xylosus* + *S. hominis* and *S. aureus* + *S. pseudintermedius* + *S. pasteruri* + *S. warneri*.

Regarding *Enterococcus*, at least one enterococcal species was found in 62.6% of the animals. The combination *E. faecium* + *E. casseliflavus* was found in three animals and *E. faecalis* + *E. gallinarum* in two animals. Moreover, three different species were detected in two animals (*E. faecium* + *E. hirae* + *E. mundtii*, and *E. faecalis* + *E. gallinarum* + *E. mundtii*) and five different enterococcal species (*E. faecium* + *E. casseliflavus* + *E. hermanniensis* + *E. hirae* + *E. mundtii*) were detected in one of the nasal samples tested (Figure 3).

### 3.4. Diversity of Gram-Negative Bacteria

Among the Gram-negative bacteria, 23 different genera were identified (Table 2). Two different *Enterobacter* species were detected in eight animals (*E. cloacae* + *E. kobei* in four animals, *E. cloacae* + *E. asburiae*, *E. bugandensi* or *E. hormaechei* in three animals, *E. asburiae* + *E. ludwigii* in one animal). Three different *Enterobacter* species were detected in one animal: *E. cloacae* + *E. hormaechei* + *E. ludwigii*. Moreover, two *Citrobacter* species (*C. gillenii* + *C. freundii* in two animals and *C. gillenii* + *C. braakii* in one) were recovered in three animals. In addition, two *Achromobacter* species (*A. spanius* + *A. xylosoxidans*) and two *Escherichia* species (*E. coli* + *E. hermannii*) were obtained in one animal each (Figure 3).

### 3.5. Co-Colonization of Bacterial Species in Nasal Samples of European Wild Rabbits

**Figure 4:**
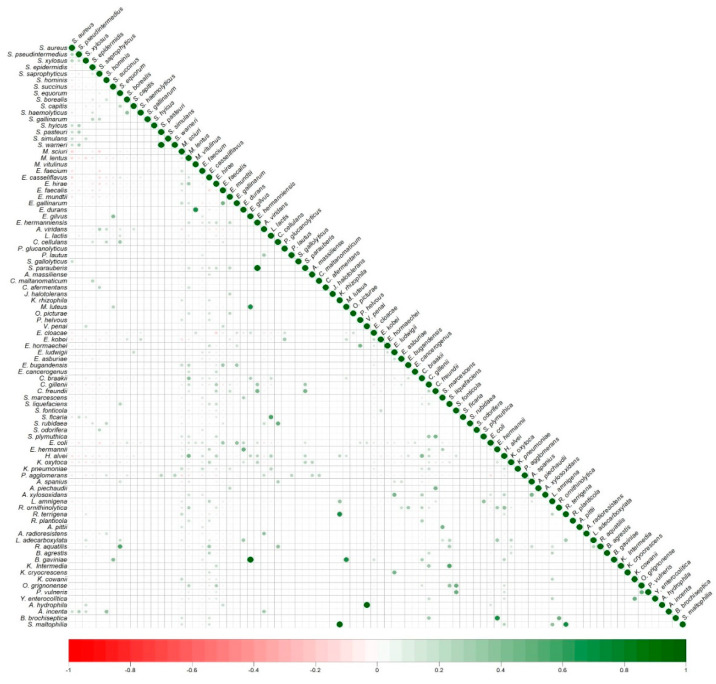
Correlation matrix of bacteria in the nasal cavities of European wild rabbits. The color and size of the circle correspond to the level of correlation: positive correlation (green) and negative correlation (red).

The correlation between the detection of the different bacterial species is shown in Figure 4. Cohabiting species with the highest positive correlation (ρ = 1) were (big green circles) one combination of two Gram-positive species (*Streptocccus parauberis* + *Enterococcus hermanniensis* and *Staphylococcus pasteuri* + *Staphylococcus warneri*) and different combinations of Gram-positive and Gram-negative species (*Buttiauxella gaviniae* + *Enterococcus gilvus*; *Aeromonas hydrophila* + *Vagococcus penai* and *Stenotrophomonas maltophila* + *Kocuria rhizophila*). A relevant positive correlation was also found for some staphylococcal species: *S. haemolyticus* + *S. borealis* (ρ = 0.40) and *S. pseudintermedius* + *S. aureus*, *S. hyicus*, *S. pasteuri* or *S. warneri* (ρ ≈ 0.30), Gram-negative species from different genera [*Raoultella terrigena* + *Kocuria rhizophila* or *S. maltophila* (ρ = 0.7/ρ = 1), *Bordetella bronchiseptica* +*Escherichia hermanii* (ρ = 0.70), and *Serratia fonticola* + *Ochrobactrum grignonense* or *Pseudescherichia vulneris* and *Bordetella bronchiseptica* + *Raoultella ornithinolytica* (ρ = 0.49)] and Gram-positive and Gram-negative species [*Staphylococcus equorum* + *Rahnella aquatilis and Lactococcus lactis* + *Serratia ficaria* (ρ = 0.57), and *Enterococcus durans* + *E. hermanii* and *Cellulosimicrobium cellulans* + *Serratia rubidarea* (ρ = 0.49)].

Although the negative correlations showed less intensity, they were detected for the enterococcal species *Enterococcus faecalis* + *Enterococcus casseliflavus* (ρ = −0.16), and among mammaliicoccal, staphylococcal or enterococcal species [*Mammaliicoccus sciuri* + *S. aureus* (ρ = −0.19), *S. aureus* + *Enterococcus casseliflavus* (ρ = −0.18), *M. sciuri* + *Staphylococcus saprophyticus* and *E. faecalis* + *E. casseliflavus* (ρ = −0.16), *E. casseliflavus* + *S. saprophyticus* (ρ = −0.14)], and even among Gram-negative and Gram-positive species *E. coli* + *S. saprophyticus* (ρ = −0.14)].

### 3.6. Phenotype of Antimicrobial Resistance

**Figure 5:**
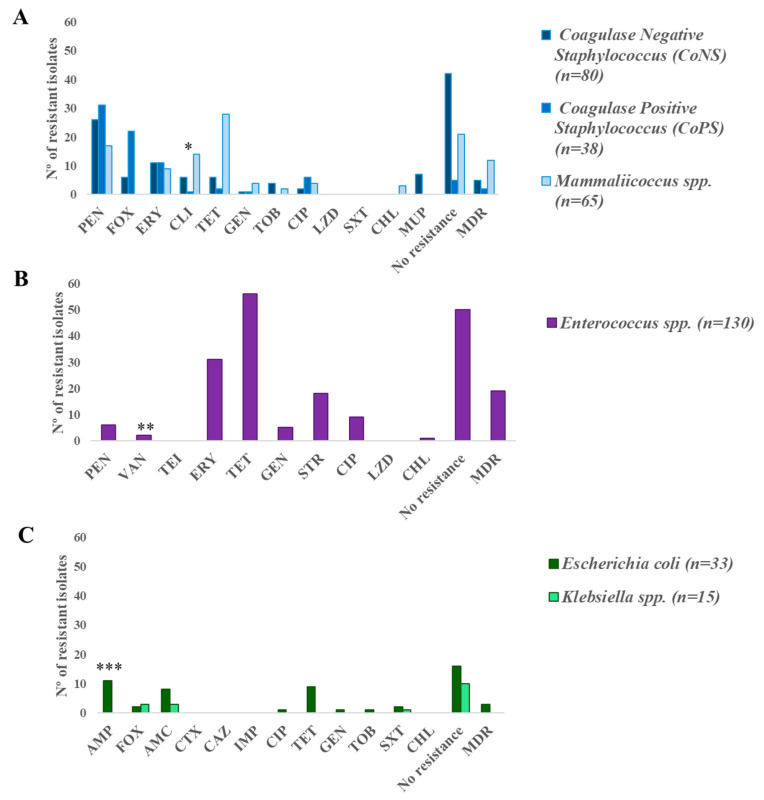
(**A**) Coagulase-negative *Staphylococcus* (CoNS), coagulase-positive *Staphylococcus* (CoPS) and *Mammaliicoccus* spp. (**B**) *Enterococcus* spp., (**C**) *Escherichia coli* and *Klebsiella* spp. PEN: penicillin; FOX: cefoxitine; AMP: ampicillin; AMC: amoxicillin–clavulanic; CTX: cefotaxime; CAZ: ceftazidime; IMP: imipenem; ERI: erythromycin; CLI: clindamycin; TET: tetracycline; CN: gentamicin, STR: streptomycin; TOB: tobramycin; CIP: ciprofloxacin, SXT: trimethoprim-sulphamethoxazole; LZD: linezolid, CHL: chloramphenicol; MUP: mupirocin; VAN: vancomycin; TEI: teicoplanin. * Intrinsic clindamycin resistance of *M. sciuri* was not considered. ** Intrinsic vancomycin resistance of *E. casseliflavus* and *E. gallinarum* was not represented. *** Intrinsic ampicillin resistance of *Klebsiella* spp. was not represented.

The AMR rates detected in staphylococci/mammaliicocci, enterococci, *E. coli*, *Klebsiella* spp. and *Y. enterocolitica* isolates are shown in Figure 5. Sixty-eight of the total staphylococci/mammaliicocci isolates (37.2%) were susceptible to all antimicrobials tested, and the remaining showed resistance to one or more antimicrobial agents: penicillin (40.5%), cefoxitin (15.3%), erythromycin (17.0%), clindamycin (11.5%), tetracycline (19.7%), gentamicin (3.8%), tobramycin (3.3%), ciprofloxacin (6.6%), chloramphenicol (1.7%), and mupirocin (3.8%). In the case of *M. sciuri*, all isolates had intrinsic resistance to clindamycin (and this resistance phenotype was not considered among AMR rates). Nineteen staphylococci of the total staphylococci/mammaliicocci (10.4%) showed a multidrug resistance phenotype (MDR) (resistance to three or more antimicrobial families) and linezolid resistant isolates were not identified. Twenty-two of the 38 CoPS isolates (57.9%) were methicillin-resistant, all of them *S. aureus* (MRSA), and they were obtained of 18/147 animals (12.2%).

Among enterococci isolates, intrinsic vancomycin resistance was detected in 15 isolates (13 *E. casseliflavus* and 2 *Enterococcus gallinarum*). Two *E. faecalis* isolates showed decreased susceptibility to vancomycin, but teicoplanin resistance was not detected. High tetracycline resistance rates (43.1%) were identified, followed by erythromycin (23.9%) and streptomycin (13.9%) resistance. Fifty isolates (38.5%) were susceptible to all antimicrobial agents, nineteen (6.9%) were MDR, and no linezolid resistant enterococci isolates were identified.

Regarding *E. coli*, almost half of the isolates (51.6%) were susceptible to all tested antimicrobial agents. In the remaining *E. coli* isolates, the following resistance rates were detected: ampicillin (35.5%), cefoxitin (6.5%), amoxicillin–clavulanic acid (25.8%), ciprofloxacin, gentamicin, tobramycin (3.3%, each one), tetracycline (29.0%) and trimethoprim–sulphamethoxazole (6.5%). In *Klebsiella* spp., in addition to ampicillin intrinsic resistance, resistance to other beta-lactams such as cefoxitin and amoxicillin–clavulanic were detected in three *K. oxytoca* isolates (23.1%). Moreover, one *K. oxytoca* isolate was trimethoprim-sulphamethoxazole-resistant. None of the *E. coli* or *Klebsiella* isolates produced extended-spectrum β-lactamases (ESBL).

The antimicrobial phenotype of the three *Y. enterocolitica* isolates obtained was also analyzed, with two of them being susceptible to all antimicrobials tested and one isolate being resistant to ampicillin.

## 4. Discussion

There is very little information about nasal microbial diversity in European wild rabbits [15]. Most studies are focused on pet rabbits and their intestinal microbiota. Moreover, in these studies, the microbiome has been mostly analyzed by genomic sequencing technologies (culture-independent approach). In these cases, *Firmicutes*, *Proteobacteria*, *Euryarchaeota*, *Tenericutes* or *Bacteroidota* phyla are usually the most predominant ones [19,26,27]. Although the majority of studies have been focused on the gut microbiome, important differences have been observed in some of them among mouth or oral parts in comparison with intestinal sites [19].

In this study, a high diversity of Gram-positive and Gram-negative genera was found. In the oral cavity of healthy pet rabbits in France, the most frequently cultivable isolated bacteria were *Streptococcus* spp. (19.8%), *Rothia* spp. (17.9%), *Enterobacter* spp. (7.0%), *Staphylococcus* spp. (6.6%) and *Actinomyces* spp. (5.7%) [28]. Although the genera detected were identified at different frequencies, in comparison with our present study, a predominance of Gram-positive genera was detected.

The predominance of *Staphylococcus* in nasal samples of European wild rabbits was not unexpected. As was corroborated by previous findings by our research group, this genus has been frequently detected in other wild animals such as small mammals, mouflon, wild boar, red deer, or several bird species [17,18,29,30]. Among staphylococci, the most common species identified was *S. aureus* (23.7% of total staphylococci), detected in 13.6% of animals analyzed (12.2% of animals carried MRSA). *Staphylococcus aureus* is an important opportunistic pathogen associated with important infectious diseases in humans and animals [31,32]. This species has also been associated with the acquisition and spread of clinically relevant AMR genes. In other studies, in which nasal and/or fecal samples of wild mammals (including rabbits) were analyzed, a similar prevalence of MRSA was detected [18,33,34]. In the present study, 15.3% of staphylococci and mammaliicocci were methicillin-resistant. For these, future studies will be carried out to determine the mechanism of AMR. Interestingly, 10.4% of staphylococci/mammaliicocci showed an MDR phenotype, and most of them were coagulase-negative *Staphylococcus* (*S. saprophyticus*, *S. epidermidis*, *S. xylosus*, *S. hominis*) or *Mammaliicoccus* species (*M. sciuri*, *M. lentus*). Coagulase-negative *Staphylococcus* and *Mammaliicoccus* appear to be important reservoirs of AMR genes that are often located on mobile genetic elements and, therefore, might be transferred to more pathogenic bacterial species [35,36].

*Enterococcus* spp. were also very prevalent, mainly the species *E. faecium*. Enterococci are among the most widely distributed genera in animal gut microbiota [37]. In one study in which rectal samples of 103 different wild mammals were analyzed, the prevalence of enterococci ranged from 0.8% to 26.4%. Curiously, the highest rate (26.4%) was found in European wild rabbits [38]. However, there is less information on its prevalence at the nasal/oral level. In nasotracheal samples of storks, the prevalence of enterococci among cultivable microbiota was 20.5% [17]. The detection of high-level contamination in nasal samples could be indicative of nasal contamination from an environment contaminated with fecal material, where the rabbits reside and feed [39]. High tetracycline resistance rates were identified among enterococci isolates, and 19 isolates were MDR. Linezolid- and vancomycin-acquired resistance has been identified previously in other wild animals such as boars, small mammals and different bird species [17,40,41,42]. In our study, two *E. faecalis* species showed decreased susceptibility to vancomycin, and none were linezolid-resistant. The absence of linezolid and vancomycin in this category of animals could be associated with less antibiotic pressure, as boars are largely and evolutionarily related to pigs (important livestock species with high AMR bacteria).

Few Gram-negative bacteria were obtained from the nasal cavities of European wild rabbits. The combination of a predominantly Gram-positive microbiota, natural defense mechanisms and a less favorable environment for Gram-negative bacteria explains their lower presence in the nasal cavities of rabbits [43,44]. Moreover, the isolates presented fewer AMR and MDR phenotypes. Worryingly, in other works, higher rates of MDR *E. coli* have been detected in wild animals [45,46,47].

Isolates producing extended-spectrum β-lactamases were not detected among our samples. However, two important pathogenic species, *Y. enterocolitica* and *B. bronchiseptica,* were identified in three animals and one animal, respectively. *Yersinia enterocolitica* is known to cause human enteric infections, but some biotypes have been related to septicemia [48]. This relevant pathogenic species has been detected previously in pets [49], but its presence in wildlife should be monitored. Regarding *B. bronchiseptica*, it has been mainly associated with respiratory diseases in humans and animals, being one of the main causes of snuffles in rabbits [50]. Although *B. bronchiseptica* infections are rare in humans, patients with compromised immunity are at increased risk of opportunistic infections with this species, and infections in healthy adults and children have also been reported [51]. It has been suggested that wild animals are involved in the epidemiology of most zoonoses [52]. According to our results, wild rabbits can be an important reservoir for the transmission of zoonotic agents to domestic animals and humans.

In this culturomics study, the nasal microbial diversity of wild rabbits was analyzed. It must be considered that a culture-dependent methodology was used. Therefore, only a fraction of the microorganisms that can grow in artificial culture media were identified, and a large part of the microbial diversity could not be detected. Future metagenomic studies could help to provide more data on the microbiota of these animals in the regions analyzed.

## 5. Conclusions

In conclusion, a high bacterial diversity was detected in nasal samples from European wild rabbits on the Iberian Peninsula, including pathogenic and/or resistant species of public health interest. A predominance of Gram-positive genera was detected, and some microbial genera or groups could act as reservoirs of important antimicrobial resistance mechanisms (coagulase-negative *Staphylococcus*, *Mammaliicoccus* or *Enterococcus*). Important pathogenic species such as *Y. enterocolitica* and *B. bronchiseptica* were detected, indicating that wild rabbits can be involved in the epidemiology of relevant zoonoses. This finding warrants close monitoring and surveillance due to the related implications for public health.

## Figures and Tables

**Table 1:** Distribution pattern of Gram-positive bacteria from nasal samples of European wild rabbits.

Genera	Species	Number of Isolates *	Number of Animals with:	
			This Species	This Genus
*Staphylococcus*	*S. aureus*	28	20	65
	*S. pseudintermedius*	10	10	
	*S. xylosus*	20	18	
	*S. saprophyticus*	14	14	
	*S. epidermidis*	13	12	
	*S. hominis*	9	6	
	*S. succinus*	7	6	
	*S. equorum*	4	4	
	*S. borealis*	3	3	
	*S. capitis*	3	3	
	*S. haemolyticus*	2	2	
	*S. gallinarum*	1	1	
	*S. hyicus*	1	1	
	*S. pasteuri*	1	1	
	*S. simulans*	1	1	
	*S. warneri*	1	1	
*Mammaliicoccus*	*M. sciuri*	34	30	45
	*M. lentus*	30	22	
	*M. vitulinus*	1	1	
*Enteroccoccus*	*E. faecium*	41	36	
	*E.casseliflavus*	26	24	
	*E. hirae*	23	20	
	*E. faecalis*	19	17	
	*E. mundtii*	13	13	92
	*E. gallinarum*	4	4	
	*E. durans*	2	2	
	*E. gilvus*	1	1	
	*E. hermanniensis*	1	1	
*Aerococcus*	*A. viridans*	9	9	9
*Lactococcus*	*L. lactis*	6	6	6
*Cellulosimicrobium*	*C. cellulans*	2	2	2
*Micrococcus*	*M. luteus*	2	2	2
*Paenibacillus*	*P. glucanolyticus*	1	1	2
	*P. lautus*	1	1	
*Streptococcus*	*S. gallolyticus*	1	1	2
	*S. parauberis*	1	1	
*Aeromicrobium*	*A. massiliense*	1	1	1
*Carnobacterium*	*C. maltanomaticum*	1	1	1
*Corynebacterium*	*C. afermentans*	1	1	1
*Jeotgalicoccus*	*J. halotolerans*	1	1	1
*Kocuria*	*K. rhizophila*	1	1	1
*Oceanobacillus*	*O. picturae*	1	1	1
*Pseudoclavibacter*	*P. helvous*	1	1	1
*Vagococcus*	*V. penai*	1	1	1

* Considering non-repetitive isolates: one isolate per species and antimicrobial resistance phenotype per animal.

**Table 2:** Distribution pattern of Gram-negative bacteria from nasal samples of wild rabbits.

Genera	Species	Number of Isolates *	Number of Animals with:	
			This Species	This Genus
*Enterobacter*	*E. cloacae*	22	22	
	*E.r kobei*	9	9	
	*E. hormaechei*	5	5	
	*E. ludwigii*	5	5	38
	*E. asburiae*	4	4	
	*E. bugandensis*	2	2	
	*E. cancerogenus*	1	1	
*Citrobacter*	*C. braakii*	11	11	
	*C. gillenii*	9	9	22
	*C. freundii*	5	5	
*Serratia*	*S. marcenses*	5	5	16
	*S. liquefaciens*	3	3	
	*S. ficaria*	2	2	
	*S. fonticola*	2	2	
	*S. rubidaea*	2	2	
	*S. odorífera*	1	1	
	*S. plymuthica*	1	1	
*Escherichia*	*E. coli*	31	25	26
	*E. hermannii*	2	2	
*Klebsiella*	*K. oxytoca*	13	12	14
	*K. pneumoniae*	2	2	
*Hafnia*	*H. alvei*	14	14	14
*Pantoea*	*P. agglomerans*	12	12	12
*Achromobacter*	*A. spanius*	5	5	6
	*A. piechaudii*	1	1	
	*A. xylosoxidans*	1	1	
*Lelliottia*	*L. amnigena*	7	7	7
*Raoultella*	*R. ornithinolytica*	4	4	7
	*R. terrígena*	2	2	
	*R. planticola*	1	1	
*Acinetobacter*	*A. radioresistens*	3	3	4
	*A. pittii*	1	1	
*Leclercia*	*L. adecarboxylata*	4	4	4
*Ranhella*	*R. aquatilis*	3	3	3
*Buttiauxella*	*B. agrestis*	2	2	3
	*B. gaviniae*	1	1	
*Yersinia*	*Y. enterocolitica*	3	2	2
*Kluyvera*	*K. cryocrescens*	1	1	2
	*K. intermedia*	1	1	
*Kosakonia*	*K. cowanii*	2	2	2
*Ochrobactrum*	*O. grignonense*	2	2	2
*Pseudescherichia*	*P. vulneris*	2	2	2
*Aeromonas*	*A. hydrophila*	1	1	1
*Advenella*	*A. incenta*	1	1	1
*Bordetella*	*B.brochiseptica*	1	1	1
*Stenotrophomonas*	*S. maltophila*	1	1	1

* Considering non-repetitive isolates: one isolate per species and antimicrobial resistance phenotype per animal.

## Data Availability

All the data derived from this study are comprehensively presented in this article. However, additional information may be requested from the corresponding author.
